# Case report of minimally invasive spinal endoscopic debridement and pedicle screw fixation for severe spinal infection of the lumbosacral spine

**DOI:** 10.1016/j.xnsj.2024.100530

**Published:** 2024-07-22

**Authors:** Vijidha Shree Rajkumar, Yingda Li

**Affiliations:** a Department of Neurosurgery, Norwest Private Hospital, 11 Norbrik Drive, Bella Vista, New South Wales 2153, Australia; b Faculty of Medicine and Health, Sydney Medical School: University of Sydney, Camperdown, New South Wales 2050, Australia

**Keywords:** Endoscopic spinal debridement, Spinal injection, Discitis, Vertebral osteomyelitis, Spondylodiscitis, Ventral phlegmon, Percutaneous pedicle screw fixation

## Abstract

**Background:**

Surgical treatment of spinal infections, refractory to medical treatments, is increasing in incidence. Here, we present a unique case of discitis secondary to an iatrogenic cause, spinal steroid injection, that resulted in acute neurology, ventral phlegmon, and osteomyelitis requiring multiple surgical interventions for treatment.

**Case Description:**

With the adoption of minimally invasive spinal surgery, the patient underwent full endoscopic debridement and decompression at our hospital. The endoscopic technique offers a unique avenue to the anatomically difficult ventral phlegmon for surgical excision, cultures, and pathogen identification. The endoscopic debridement was paired with percutaneous pedicle screw fixation to stabilize the spine from the worsening bone destruction.

**Outcome:**

The patient recovered well postoperatively, with the resolution of her neurological symptoms and improved mobility.

**Conclusions:**

Full endoscopic spinal debridement and decompression is a powerful tool to manage severe spinal discitis and preliminary studies encourage its adoption in surgical practices.

## Introduction

Spinal steroid injections are commonly used as diagnostic and therapeutic spinal interventions before surgeries for disc pathology in the lumbar spine (L-spine). Complications from this procedure, although infrequent, include procedural site infections, which have an estimated incidence of 1% to 2 % [[1], [2], [3]]. Even rarer are the more severe infections such as epidural abscesses, osteomyelitis, and discitis, with an estimated incidence of 1 to 2 cases per 10,000 patients [4]. When they do occur, however, the morbidity from these infections can be severe, requiring immediate medical attention. Once refractory to medical treatments, these patients may require surgical interventions for pathogen identification, and debridement for source control. Additionally, worsening spinal infections necessitate neural decompression, instrumentation, or both, to prevent further neurological deterioration and provide spinal stability [5]. Varied approaches have been described for the management of spondylodiscitis. These include anterior, posterior, and combined open surgery, plus more recently, minimally invasive spinal surgery with endoscopic debridement and drainage [[6], [7], [8]]. Here, we present a rare case of severe spinal infection secondary to spinal steroid injection that required multiple surgical interventions and prolonged medical care for recovery.

## Methods

### Case report

#### History

**Fig. 1:**
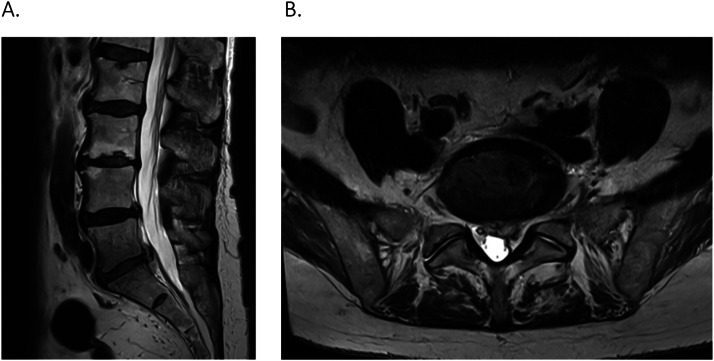
(A) T-2 weighted MRI L-spine on sagittal view demonstrates the patient's initial disc budge at L5/S1. (B) T-2 weighted MRI L-spine on axial view of the L5/S1 disc with left paracentral disc protrusion and compression of left S1 nerve root.

A 69-year-old female presented with worsening back pain, 1-week post nerve root injection for left S1 nerve root compression from L5/S1 disc protrusion (Fig. 1). She has a known medical history of paroxysmal atrial fibrillation, hypothyroidism, essential thrombocythemia, and asthma.

Medications: Verapamil, Aspirin, Thyroxine, Digoxin

Allergy: Codeine

#### Examination and investigations

**Fig. 2:**
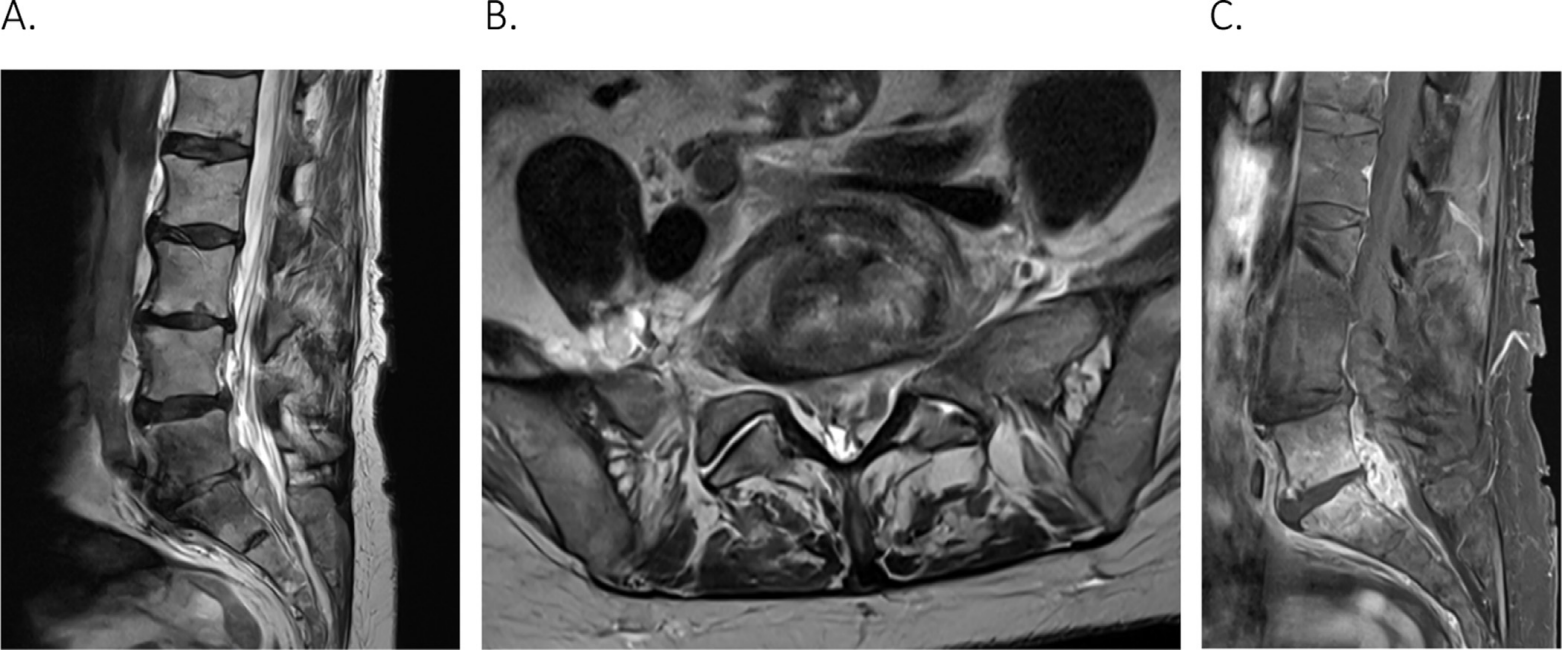
(A) T-2 weighted MRI L-spine on sagittal view demonstrates extensive inflammatory changes centered at the L5/S1. (B) T-2 weighted MRI L-spine on axial view showing mass effect with compression of the thecal sac, epidural material is seen extending into the exit foramina. (C) Postcontrast T-1 weighted MRI L-spine on sagittal view illustrates an extensive enhancing inflammatory phlegmon in the ventral epidural space extending from L4/5 to S1/2, approximately 43 mm in craniocaudal length.

On examination, the patient had lumbar back pain with radiation down to the posterior aspect of the left leg. She had associated numbness on the lateral side of the left foot and reduced power with left plantar flexion (power 4+/5). She did not report any saddle anesthesia, or bowel and bladder dysfunction. The remainder of her neurological examination was unremarkable. The patient denied any systemic symptoms of an infection; however, she had elevated inflammatory markers and leucocytosis with white cell count (WCC) of 11.8×10*9/L, neutrophils of 8.3×10*9/L, and C-reactive protein (CRP) of 126 mg/L. On imaging, the magnetic resonance imaging (MRI) of the L-spine showed inflammatory changes at L5/S1 consistent with discitis, an enhancing phlegmon in the ventral epidural space extending from L4/5 to S1/2, and compression of the thecal sac at the S1 level (Fig. 2).

**Fig. 3:**
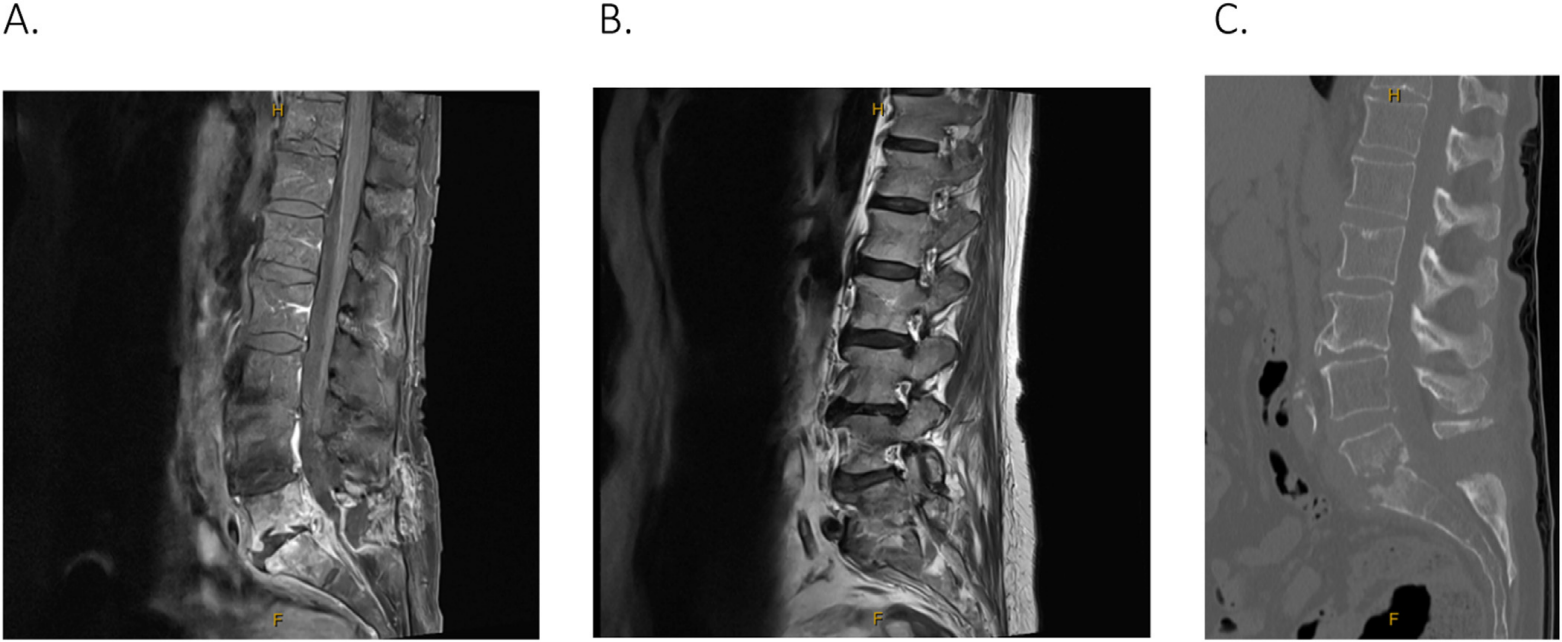
(A) Postcontrast T-1 weighted MRI L -spine on sagittal view illustrates further reduction in disc space height, worsening irregularity of the endplates, and mass effect on the thecal sac due to the epidural collection. (B) T-2 weighted MRI L-spine on sagittal view showing mass effect residual compromise of the left exit foramina. (C) CT L-spine, bone window, on sagittal view illustrates worsening bone destruction at the L5/S1 level.

She was urgently taken to the operation theatre for L5/S1 laminectomy and decompression plus washout of the epidural abscess. Intraoperatively, there was severe spinal stenosis secondary to the phlegmon and moderate left paracentral disc herniation, but no frank pus was seen. Multiple samples were taken for cultures from the ventral epidural phlegmon; however, nil organisms were isolated. Based on local Infectious Diseases’ advice, the patient was commenced on intravenous (IV) Vancomycin and Tazocin with close clinical monitoring to assess response to medical treatment. Despite this, along with acute pain services to manage analgesia requirements including IV ketamine, the patient developed worsening mechanical lumbar back pain, left L5 radicular pain, and immobility over the ensuing month. The physiotherapy assessment revealed that the patient, initially independent with mobility on admission, required a forearm support frame with 2 moderate assists to mobilize on the wards. The repeat scans showed further bone destruction, progression of discitis at L5/S1 disc space, persistent epidural enhancement of phlegmon, and worsening left foraminal stenosis (Fig. 3). On the hematological screen, the patient had raised inflammatory markers with WCC of 14.8×10*9/L, neutrophils of 13.9×10*9/L, and CRP of 97.3mg/L.

Given the patient had a progression of spondylodiscitis clinically, with worsening pain and mobility, a variety of surgical approaches were discussed, and the patient consented to a minimally invasive approach with instrumentation. The patient was recommended a transforaminal endoscopic surgery to decrease the burden of disease from direct debridement of the ventral phlegmon plus irrigation, dilution and egression of the disc space, and decompression of the nerve. Furthermore, the patient required stabilization of the L5/S1 level due to worsening bone destruction affecting her mobility and function. Given the poor bone quality at the level of infection, the patient was recommended to have a longer construct - L3 to pelvis posterior fusion - to help stabilize and fuse the spine. The patient underwent a Left L5/S1 transforaminal endoscopic foraminotomy through trans–SAP (superior articular process) approach, debridement of epidural phlegmon, and percutaneous L3-iliac fixation.

#### Operation

**Fig. 4:**
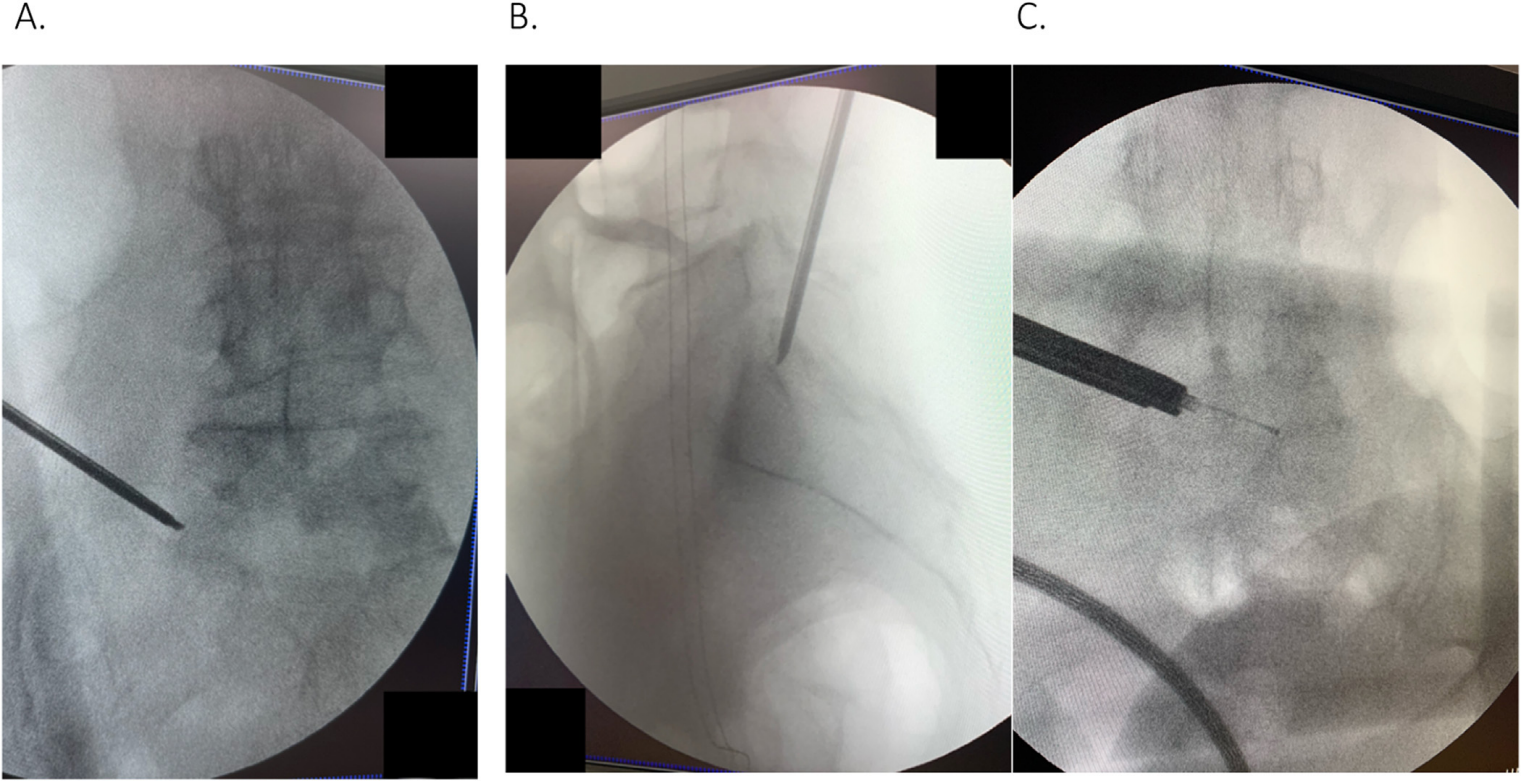
(A) Intraoperative fluoroscopy image to confirm targeting of left sacral superior anticircular process. (B) Intraoperative fluoroscopy image with left L5/S1 foramen drilled and expanded to decompress and the exiting and traversing nerve root. (C) Intraoperative fluoroscopy image post ventral phlegmon excision displaying large epidural void and easy passage of blunt probe past midline.

**Fig. 5:**
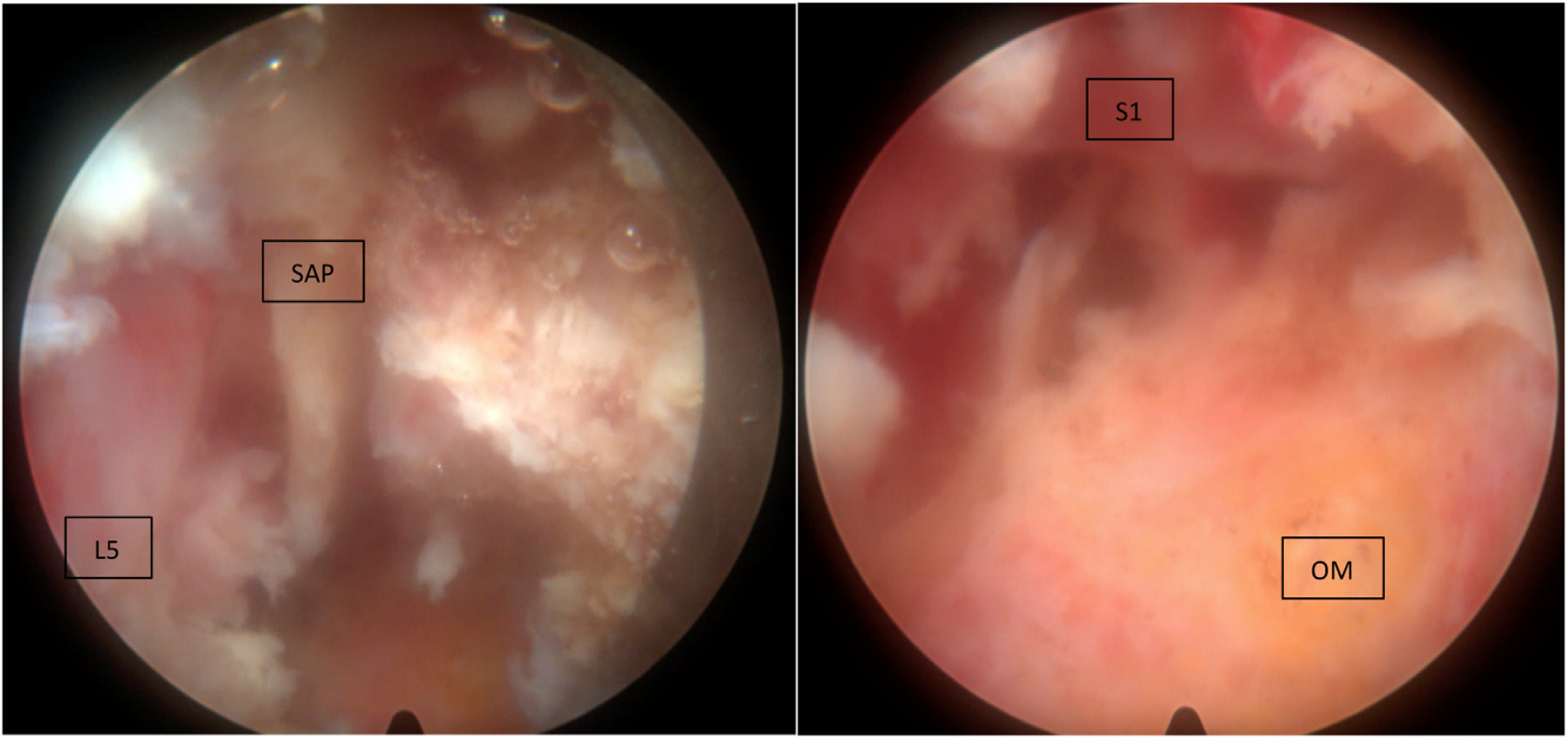
(A) Endoscopic view of the drilled tip of the SAP and adjacent cancellous bone to expand the left exit foramen and decompression of the L5 nerve root. (B) Endoscopic view of decompressed S1 nerve root from the debrided disc space at the center of the screen and adjacent spondylodiscitis (OM) appearing yellow.

Following prone positioning on the Wilson frame with the Jackson table, percutaneous targeting of the left S1 superior articular process (SAP) with a Jamshidi needle was performed under fluoroscopy. This was followed by the sequential guide wire, dilators, and reamers to expand the entry channel for the beveled retractor and endoscope (Joimax TESSYS) into the left L5/S1 intervertebral space. The foramen was expanded by drilling of SAP and rostral S1 pedicle, followed by piecemeal excision of the ventral phlegmon with pituitary forceps (Fig. 4A, 4B). There was easy passage of the probe past the midline in the ventral aspect of the canal upon expansion of the intervertebral foramen (Fig. 4c). The L5 exiting and traversing S1 nerve roots were directly decompressed with confirmation of easy passage of the probe across the ventral epidural space (Fig. 5). The patient had bilateral L3 and L4 percutaneous pedicle screws inserted under fluoroscopic guidance, followed by insertion of bilateral iliac screws through her existing midline incision and connected with rods.

**Fig. 6:**
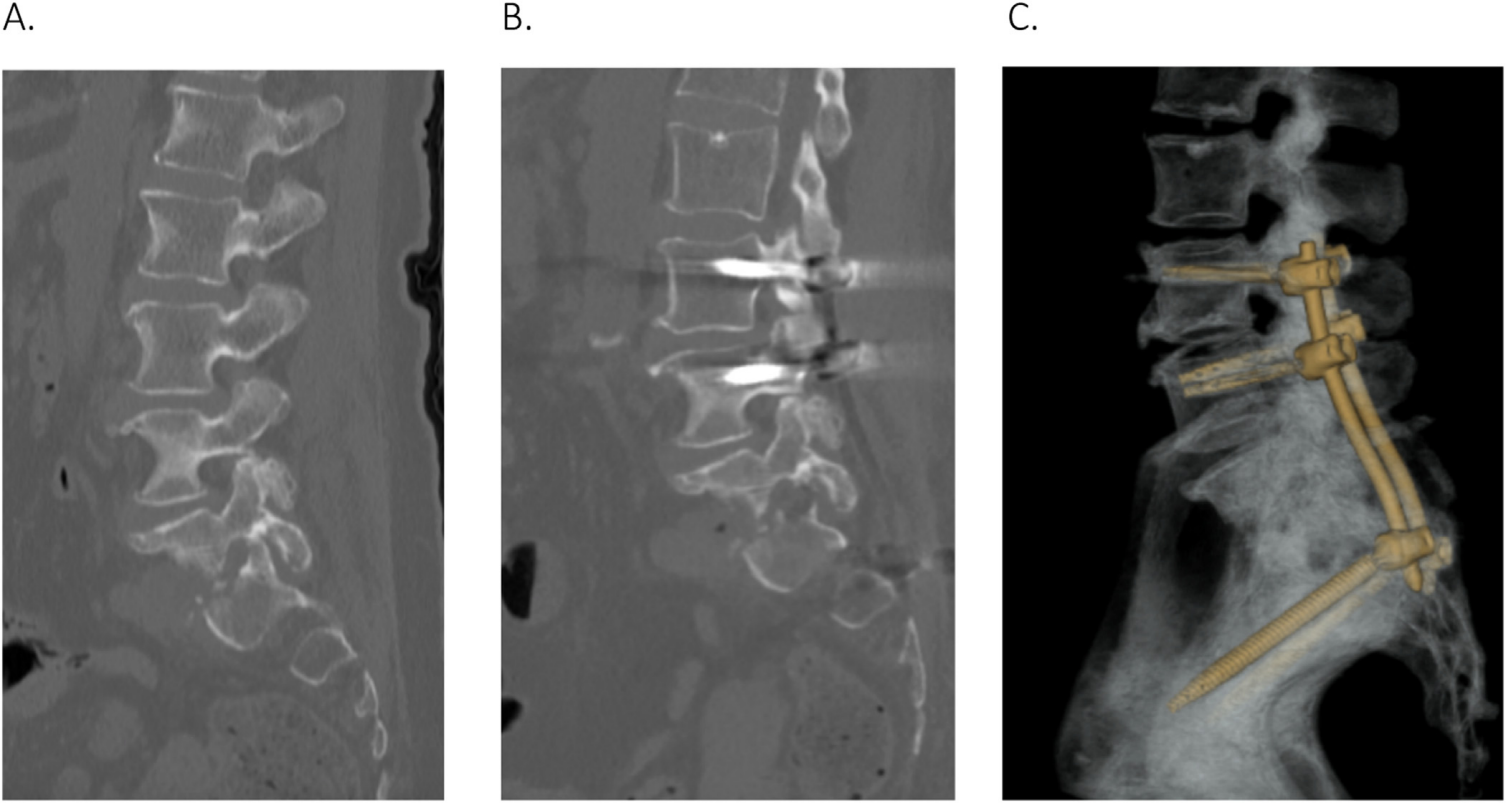
(A) – Pre-op CT L-spine, bone window, on sagittal view illustrates narrowing of L5/S1 left foramen. (B) Post-op CT L-spine, bone window, on sagittal view illustrates decompression of the left L5/S1 foramen. (C) Post-op 3-dimensional reconstruction of the construct.

Postoperative CT L-spine confirmed adequate foraminal decompression and satisfactory position of the hardware (Fig. 6).

## Discussion and conclusion

Discitis and vertebral osteomyelitis are increasing in incidence [9]. Here, we presented a case of severe spinal infection, secondary to spinal steroid injection, refractory to medical treatment, and posterior surgical decompression. With the increasing adoption of minimally invasive spinal surgery across the globe, we highlight the option of endoscopic debridement as an alternative to open surgery in the management of spinal infections. Our patient was initially commenced on intravenous antibiotics for medical management of her spinal infection. Studies have supported the empirical use of vancomycin in spinal epidural abscesses, given most cases are caused by Gram-positive cocci [10]. However, our patient had progression of the infection, without pathogen isolation for cultures and sensitivities, prompting further tissue sampling for diagnosis. Endoscopic debridement and drainage allow for tissue sampling under direct vision for cultures. This technique has also been shown to have higher rates of pathogen identification in comparison to needle biopsies [11]. Comprehensive local debridement of refractory infections increases its resolution rate in cases of spondylodiscitis [12,13].

Studies have shown multiple predisposing factors associated with patients who fail medical therapy. These include diabetes mellitus, immunosuppression, persistently raised inflammatory markers, and progression of neurology [[14], [15], [16], [17], [18]]. Other social factors associated with refractory infections that require surgical intervention include alcohol misuse, intravenous drug use, and obesity [10]. Among these, our patient had obesity, persistently raised inflammatory markers despite medical treatment, and progression of neurology, which ultimately required surgical intervention for treatment.

Multiple endoscopic approaches to the lumbosacral spine have been described extensively elsewhere to address spinal pathology [19,20]. This includes transforaminal, trans-SAP, interlaminar, and biportal endoscopic techniques [[21], [22], [23]]. Transforaminal endoscopy offers a distinct and direct approach to decompress the neural foramen [24]. Conversely, the trans-SAP approach provides a bony landing zone instead of traversing through soft tissue, potentially reducing the incidence of dorsal root ganglion (DRG) irritation [25]. This benefit may be more pronounced at the caudal lumbar levels where the height of the foramen decreases, while the size of the DRG increases. The trans-SAP approach was recommended for this patient to decompress the nerve, and expand the left intervertebral foramen, providing direct access to the ventral aspect of the spinal canal to debride the phlegmon. The positive inflow from the endoscope, constant irrigation, and egression both debride and dilute the affected space for drainage and washout [26,27]. A comparison of recent studies encourages early percutaneous endoscopic debridement and drainage for spondylodiscitis, which still need to be validated in larger studies [28].

Additionally, the patient's persistent ventral phlegmon is difficult area to address solely through open posterior debridement. The endoscopic technique, however, allows direct anatomical access to the phlegmon for surgical excision. Endoscopic approaches have been described to treat ventral abscesses in other areas of the spine as well, including the cervical spine (C-spine), thoracic spine (T-spine), and L-spine [[29], [30], [31], [32]]. These studies highlight different techniques to address infectious ventral spinal pathology expanding multiple spinal levels. Particularly, Akbik et al. [31], described successful treatment of T5-L5 ventral epidural abscess via single T9/10 transforaminal endoscopic drainage. The advantage of early endoscopic debridement is the ability to dilute purulent pus with irrigation, with a smaller incision site, plus localized debridement under direct visualization. In addition, this can be combined with percutaneous catheter placement for ongoing drainage of abscesses.

Generally, minimally invasive surgery offers added benefits for higher-risk patients including decreased anesthetic time, and the ability to perform surgery with sedation if required, while minimizing tissue damage and wound healing requirements [12,33,34]. Although full endoscopic debridement for spondylodiscitis has its advantages, there are limitations to the procedure that need to be considered. Endoscopic spine surgery has a steep learning curve, even steeper with the transforaminal approach, which requires formal training for surgeons wishing to incorporate it into their practice [35]. Furthermore, the overall incidence of complications associated with the transforaminal approach is greater than the interlaminar approach [36,37]. The more common complications reported are transient neurological deficits and dysesthesias from irritation to the nerve or DRG [35,37]. Although infrequent, there have been cases of pyogenic spondylodiscitis after endoscopic procedures [38,39]. Thus, stringent care must be taken to ensure sterile prepping, draping, double gloving, and meticulous technique when introducing instruments into the working channel during the procedure [38].

Lastly, when patients need stabilization of spinal segments due to bone destruction from the infection, full endoscopic discectomy and debridement can be paired with percutaneous pedicle screw fixation to provide stability in a minimally invasive approach [30,40]. Liu et al. [30] reported a retrospective study with seventeen patients who underwent anterior cervical debridement with posterior fixation of C-spine secondary to aggressive tuberculosis infection. Literature has shown stabilizing the affected spinal levels can accelerate healing and fusion of the spine [33,34]. Historically, instrumentation of the spine with active infective processes has been debated however, studies have highlighted its importance for the stabilization of the spine [41,42]. Moreover, titanium pedicle screws for fixation are safe and effective in pyogenic spondylodiscitis [43,44]. Our patient reported severe pain limiting mobility that required fixation to stabilize the affected segments. The procedure enabled our patient to become ambulatory postoperatively with significant pain improvement.

The patient's analgesia requirements lessened and weaned over the ensuing weeks on the wards. From a hematological perspective, the patient's inflammatory markers were within normal limits on the day of discharge with WCC of 9.9×10*9/L, neutrophils of 4.4×10*9/L, and CRP of 9.5mg/L. She subsequently recovered in the peri-operative period from a clinical, hematological, and mobility perspective after a period of rehabilitation and the patient was successfully discharged home.

At the 6-month follow-up, the patient is independently mobile with no neurologic sequelae, weaned off all analgesia, and had repeat scans showing near complete resolution of her epidural phlegmon. At her recent 1-year follow-up, the patient has radiological evidence of fusion, and she reports being back to baseline mobility, walking independently, and recovering well from her protracted hospital stay.

## Informed patient consent

Complete written informed consent was obtained from the patient for the publication of this study and accompanying images.

## Ethical approval

This study is a retrospective analysis of 1 clinical case and is exempt from ethical approval.

## Declarations of competing interests

The authors declare that they have no known competing financial interests or personal relationships that could have appeared to influence the work reported in this paper.

## Appendix Supplementary materials

Image, application 1
