# Supplementary material for: Case report of minimally invasive spinal endoscopic debridement and pedicle screw fixation for severe spinal infection of the lumbosacral spine
Source: N Am Spine Soc J. 2024 Jul 22;19:100530. doi: 10.1016/j.xnsj.2024.100530 (PMC11381435; doi:10.1016/j.xnsj.2024.100530)
Supplement: Supplementary file 1 [file mmc1.docx]

140-character tweet for promotion of work:

**Rare case of spondylodiscitis from steroid injection successfully treated with full endoscopic debridement and posterior pedicle screw fusion**
